# Cross-sectional analysis of the association between serum calcium levels and lipid profiles and the risk of cardiovascular disease in Saudi women

**DOI:** 10.1097/MD.0000000000044947

**Published:** 2025-10-17

**Authors:** Walaa Mohammedsaeed, Amal Mohammed Q. Surrati, Asma Alshanqiti

**Affiliations:** a Clinical Laboratory Sciences Department, College of Applied Medical Science, Taibah University, Medinah, Saudi Arabia; b Family, Community Medicine and Medical Education Department, College of Medicine, Taibah University, Medinah, Saudi Arabia.

**Keywords:** calcium, cardiovascular risk, lipid profile, postmenopausal, premenopausal, Saudi women

## Abstract

Calcium (Ca) plays a critical role beyond bone health, influencing lipid metabolism and cardiovascular risk. This study aimed to evaluate the association between serum Ca levels and lipid profiles among Saudi women, considering menopausal status. A cross-sectional study was conducted at Taibah University, Madinah, including 300 women aged 20 to 60 years. Participants were divided into premenopausal (n = 180) and postmenopausal (n = 120) groups. Fasting blood samples were collected to measure serum Ca, phosphorus, parathyroid hormone (PTH), vitamin D (Vit-D), and lipid profiles. Data were analyzed using analysis of variance and Pearson correlation. Postmenopausal women exhibited significantly higher levels of total cholesterol (TC), triglycerides (TG), Ca, and PTH, and lower HDL-C and Vit-D levels compared to premenopausal women (*P* < .05). Serum Ca positively correlated with TC (*R* = 0.643) and TG (*R* = 0.731) and negatively with HDL-C (*r* = –0.513) in postmenopausal women. A weaker but significant positive correlation between Ca and TC was observed in premenopausal women. Elevated serum Ca levels are associated with adverse lipid profiles, particularly in postmenopausal Saudi women, indicating an increased cardiovascular risk. Early screening and careful management of Ca and lipid levels are recommended to mitigate future cardiovascular complications.

## 1. Introduction

Calcium (Ca) is an essential nutrient known primarily for its role in bone and dental health. However, its effects extend beyond the skeletal system, influencing vascular health, insulin resistance, and lipid metabolism.^[1–3]^ Elevated serum Ca levels have been associated with metabolic syndrome and adverse lipid profiles, including hyperlipidemia, a major risk factor for cardiovascular disease (CVD).^[4]^

Ca regulates key enzymes involved in lipid metabolism, such as lipoprotein lipase and hormone-sensitive lipase.^[5]^ Disruptions in Ca homeostasis can impact lipid synthesis and degradation pathways. High serum Ca levels are linked to elevated total cholesterol (TC) and low-density lipoprotein cholesterol (LDL-C),^[6]^ potentially mediated by parathyroid hormone (PTH) interactions affecting liver function and adipocyte metabolism.^[7]^

Postmenopausal women undergo significant hormonal changes, particularly a decline in estrogen, that negatively affect lipid metabolism.^[8]^ Estrogen normally enhances HDL-C and reduces LDL-C levels; its deficiency leads to dyslipidemia. Vitamin D (Vit-D) deficiency, prevalent among postmenopausal women, further exacerbates Ca and lipid metabolism disturbances.^[9,10]^ Additionally, excessive Ca supplementation, often recommended to prevent osteoporosis, can unintentionally raise cardiovascular risks when not properly monitored.^[9]^

Recent meta-analyses and cohort studies have reinforced the link between serum Ca levels and lipid disturbances.^[11]^ However, the relationship is complex, influenced by hormonal, genetic, dietary, and lifestyle factors.

Globally, health policies recommend careful Ca supplementation, particularly in aging populations, to balance the benefits on bone health against potential cardiovascular risks. Nevertheless, research specifically addressing these associations in Saudi females remains limited. Given regional differences in diet, sun exposure, and lifestyle,^[12,13]^ investigating this population is crucial.

Thus, we conducted a cross-sectional study involving female students and staff at Taibah University in the Madinah region to evaluate the association between serum Ca levels and lipid profiles, particularly considering menopausal status.

## 2. Materials and methods

### 2.1. Study design and population

This cross-sectional study was conducted at Taibah University (Madinah, Saudi Arabia) from January 2020 to January 2022. It included 300 women aged between 20 to 60 years, consisting of both premenopausal and postmenopausal individuals. Participants were recruited through campus-wide announcements, posters, and voluntary participation. The age range of 20 to 60 years was selected to capture women from early adulthood through the menopausal transition, as hormonal changes across these stages significantly impact Ca and lipid metabolism. A systematic random sampling method was applied, where various colleges, centers, and administrative units were used as strata to ensure broad representation across the university population.

### 2.2. Sample size calculation

A priori power analysis was performed using G*Power software v3.1 (https://stats.oarc.ucla.edu/other/gpower/).

Parameters were: Medium effect size (Cohen *d* = 0.5); Significance level (α) = 0.05; Power (1−β) = 0.80.

The sample size formula used was:


$$\begin{array}{l} {\text{n}=\;\;\;(Z\alpha /2\;\;\;+Z\beta )}^{\text{2}}\times 2\times \sigma^{\text{2}}/\Delta^{\text{2}} \end{array}$$

where σ = standard deviation and Δ= mean difference between groups.

Thus, at least 50 participants per group were required; 300 participants were recruited to ensure adequate statistical power and allow subgroup analysis.

### 2.3. Inclusion criteria

Participants were eligible if they met the following criteria: Female aged 20 to 60 years; Either premenopausal or postmenopausal status confirmed via self-reported menstrual history; Willingness to report any current Ca or Vit-D supplements use, regardless of physician prescription status.

### 2.4. Exclusion criteria

Participants were excluded if they: We are currently using lipid-lowering medications; We are pregnant or breastfeeding; They had known metabolic disorders such as diabetes mellitus, thyroid dysfunction, renal failure, or malabsorption syndromes; They had a history of CVD; and They had clinically diagnosed Ca or Vit-D deficiency requiring medical treatment.

### 2.5. Specimen collection and biochemical markers analysis

Participants were instructed to fast overnight for at least 8 hours. Three milliliters (3 mL) of venous blood was collected from each participant. The blood samples were centrifuged at 3000 rpm for 5 minutes to separate the serum.

The following biomarkers were analyzed: Lipid profile: TC, high-density lipoprotein cholesterol (HDL-C), LDL-C, and triglycerides (TG) were measured enzymatically using Roche Cobas analyzers; Serum Ca and phosphorus: Measured by a colorimetric method using Roche Diagnostics kits;Vit-D and PTH levels: Determined using immunoassay technology (Cobas b 311).

Inter-assay and intra-assay coefficients of variation (CV) for these tests were within acceptable laboratory standards.

The inter-assay variability, representing the consistency of results across different runs of the assay, and the intra-assay variability, indicating the consistency within the same run, were as follows: Ca: Inter-assay CV = 3.2%, Intra-assay CV = 2.1%; PTH: Inter-assay CV = 4.5%, Intra-assay CV = 3.0%; Vit-D: Inter-assay CV = 5.0%, Intra-assay CV = 4.2%; TC: Inter-assay CV = 2.8%, Intra-assay CV = 2.0%; TG: Inter-assay CV = 3.1%, Intra-assay CV = 2.5%; HDL-C: Inter-assay CV = 3.3%, Intra-assay CV = 2.4%; and Phosphorus (P): Inter-assay CV = 2.9%, Intra-assay CV = 2.1%.

### 2.6. Classification of lipid disturbances and risk assessment

Lipid profile results were classified according to: American Heart Association criteria for TG^[14]^; National Cholesterol Education Program (NCEP) guidelines for cholesterol and LDL-C levels.^[15]^

Below are the classifications and their references: TG: According to the American Heart Association, triglyceride levels are classified as follows: Normal: <1.70 mmol/L, Borderline high: 1.70–2.25 mmol/L, High: 2.26–5.64 mmol/L^[12]^; TC: According to the NCEP Expert Panel, 2002 and European Society of Cardiology/European Atherosclerosis Society guidelines.^[13,14]^ Normal: <5.17 mmol/L, Borderline high: 5.17–6.20 mmol/L, High: >6.21 mmol/L; LDL-C as outlined by the NCEP guidelines^[13,14]^: Low risk: <3.3 mmol/L, Moderate risk: <2.6 mmol/L, High risk (e.g., patients with CVD): <1.8 mmol/L (Grundy et al,^[13]^ 2018); PTH Interpretation: PTH levels were classified based on reference ranges from the laboratory performing the analysis. Normal PTH levels typically range from 10 to 65 pg/mL. Elevated PTH levels can indicate primary hyperparathyroidism or secondary hyperparathyroidism, often due to Vit-D deficiency or chronic kidney disease; Ca and phosphorus levels: Ca: Normal serum Ca levels: 2.15–2.55 mmol/L, Hypercalcemia: >2.55 mmol/L, Hypocalcemia: <2.15 mmol/L,^[15]^ Phosphorus: Normal serum phosphorus levels: 0.8–1.5 mmol/L, Hyperphosphatemia: >1.5 mmol/L, Hypophosphatemia: <0.8 mmol/L.^[16]^

The atherogenic index of plasma (AIP) was calculated as:


$$\begin{array}{l} AIP=log_{10}(TG/HDL-C) \end{array}$$

AIP values were interpreted as: <0.11 = low cardiovascular risk; 0.11–0.21 = intermediate cardiovascular risk; 0.21 = high cardiovascular risk.^[17]^

The weight and height of the women were measured twice using a digital scale (Beurer GmbH Type PS 07, China) to determine their body mass index (BMI). Individuals were classified into different categories based on their BMI: underweight (BMI < 18.5 kg/m^2^), normal weight (BMI between 18.5–24.9 kg/m^2^), overweight (BMI between 25.0–29.9 kg/m^2^), or obese (BMI ≥ 30.0 kg/m^2^).^[18]^

### 2.7. Statistical analysis

Data were collected and statistically assessed using Graphpad Prism 7 (GraphPad Software, San Diego). Descriptive statistics were calculated for all variables, and data are presented as means ± standard deviations (SD) for continuous variables. For the comparison of means between multiple groups, an analysis of variance (ANOVA) test was used. The ANOVA test was employed to compare the means of serum Ca levels, lipid profiles, and other biochemical markers across multiple groups (premenopausal vs postmenopausal women). The ANOVA test is particularly useful for determining whether there are any statistically significant differences between the means of 3 or more independent (unrelated) groups. Post hoc tests (Tukey HSD) were conducted following significant ANOVA results to identify specific group differences. We acknowledge the unequal group sizes (180 premenopausal vs 120 postmenopausal). However, this reflects the natural recruitment flow and availability within the university population. While group imbalance may influence statistical power, appropriate statistical methods (e.g., two-way ANOVA) were used to minimize bias. Two levels of significance were utilized in this study: 0.05 and 0.001. These levels are widely accepted in biomedical research for hypothesis testing and are used to indicate that the findings are statistically significant with 95% and 99% confidence levels respectively. Pearson Correlation Coefficients: these were calculated to assess the strength and direction of the linear relationship between serum Ca levels and lipid profiles. The level of significance applied was 0.05 or 0.001 levels.

## 3. Results

### 3.1. Participant characteristics

The whole group under consideration consisted of 300 women, namely 180 premenopausal women and 120 postmenopausal women. The age range spanned from 20 to 60 years. The mean age ± SD was 33 ± 10.3 years in premenopausal women and 54 ± 10.5 years in postmenopausal women. The difference in age between pre- and postmenopausal women was statistically significant (*P* < .05). Approximately 5.5% of postmenopausal women in this study used Ca + vitamin C tablets routinely without seeing a physician. However, specific data on their regular usage is not provided. The study presented the blood lipids, serum Ca, PTH, P, Vit-D, BMI, and AIP levels of the individuals in Table 1. Postmenopausal women had elevated levels of TC, TG, PTH, and Ca, while demonstrating a decreased level of HDL-C and phosphorus (P) with low Vit-D. The AIP value was 0.11, indicating an intermediate risk of CVD in postmenopausal women.

**Table 1:** Clinical and biochemical parameters of subjects.

Parameter	Premenopausal (n = 180)	Postmenopausal (n = 120)	*P*-value
LDL-C (mmol/L)	2.31 ± 1.2	2.50 ± 1.1	>.05
HDL-C (mmol/L)	1.56 ± 0.7	1.22 ± 0.5	.05*
Total cholesterol (mmol/L)	4.10 ± 1.2	5.51 ± 1.4	.03*
Triglycerides (TG) (mmol/L)	1.5 ± 0.4	1.73 ± 0.7	.03*
Calcium (Ca) (mmol/L)	2.30 ± 0.6	2.77 ± 0.8	.05*
Phosphorus (P) (mmol/L)	0.87 ± 0.8	0.70 ± 0.7	.04*
Parathyroid hormone (PTH) (pg/mL)	50.5 ± 9.3	46.5 ± 10.4	.03*
Vitamin D (Vit-D) (nmol/L)	37 ± 10.5	20 ± 5.3	.02*
AIP	0.1 ± 0.1	0.11 ± 0.1	.05*
BMI (kg/m^2^)	23.5 ± 8.2	26.9 ± 7.3	.03*

Values were Mean ± standard deviation. References range acquired from Madinah Hospital labs in the Madinah region, Saudi Arabia. ANOVA test was employed to compare between 2 groups. Vit-D deficiency is defined as <25 nmol/L. AIP values 0.11–0.21 indicate intermediate cardiovascular risk. BMI between 25–29.9 kg/m^2^ indicates an overweight status.

AIP = atherogenic index of plasma, BMI = body mass index, Ca = calcium, HDL-C = high-density lipoprotein cholesterol, LDL-C = low-density lipoprotein cholesterol, P = phosphorus, PTH = parathyroid hormone.

* *P*-value ≤.05 was considered significant.

### 3.2. Key findings

The study included 300 women: 180 premenopausal (60%); and 120 postmenopausal (40%).

The age range was 20 to 60 years: Premenopausal mean age: 33 ± 10.3 years; Postmenopausal mean age: 54 ± 10.5 years; (*P* < .05, statistically significant difference).

Approximately 5.5% of postmenopausal women reported regular use of Ca and vitamin C tablets without medical consultation.

### 3.3. Stratification by calcium levels

Women were categorized into 3 groups based on serum Ca: Low Ca: < 2.15 mmol/L; Normal Ca: 2.15–2.55 mmol/L; and High Ca: > 2.55 mmol/L.

Table 2 presents the findings of a two-way ANOVA that compared the 3 groups. Among postmenopausal women, all measured parameters (PTH, BMI, and AIP) showed a strong and highly significant increase with increasing Ca levels.

**Table 2:** Serum calcium categories and parameters.

Parameter	Low calcium	Normal calcium	High calcium	*P*-value
Premenopausal women				
Number (%)	40 (13.3%)	125 (41.7%)	15 (5%)	–
Age (yr)	33 ± 11.3	37 ± 9.2	38 ± 11.1	>.05
PTH (pg/mL)	23.41 ± 12.3	40.53 ± 13.5	50.92 ± 11.9	.04*
AIP	0.10 ± 0.08	0.11 ± 0.09	0.12 ± 0.1	>.05
BMI (kg/m^2^)	22.5 ± 6.1	23.7 ± 6.2	23.8 ± 6.7	>.05
Postmenopausal women				
Number (%)	10 (3.3%)	50 (16.7%)	60 (20%)	–
Age (yr)	53 ± 9.5	54 ± 10.4	57 ± 12.2	>.05
PTH (pg/mL)	20.52 ± 7.4	25.51 ± 7.7	45.60 ± 10.4	.01*
AIP	0.10 ± 0.03	0.10 ± 0.05	0.12 ± 0.1	.003**
BMI (kg/m^2^)	23.9 ± 7.1	24.1 ± 7.2	25.7 ± 8.4	.05*

Data were indicated as the mean ± (SD) or number (%) for continuous variables, *P*-value obtained from two-way ANOVA.

AIP = atherogenic index of plasma, BMI = body mass index, PTH = parathyroid hormone.

* *P* ≤ .05,

** *P* ≤ .001.

### 3.4. Stratification by cholesterol levels

In addition, we categorized women (both pre- and postmenopausal) into 3 groups based on their TC levels (Normal < 5.17mmol/L, Borderline 5.17–6.20, High ≥ 6.21mmol/L). The findings of the two-way ANOVA analysis can be found in Table 3. Notably, the levels of Ca in the blood serum showed a substantial and steady rise from group 1 to group 3 in both pre- and postmenopausal women (all *P* < .05). In postmenopausal women, the AIP value showed a significant rise (*P* = .002) from group 1 to group 3. However, there was no difference in the AIP value across the groups in premenopausal women.

**Table 3:** Serum calcium of women (pre- and postmenopausal) separated relating to triglyceride values.

Parameter	Normal < 1.70 mmol/L	Borderline high 1.70–2.25 mmol/L	High 2.26 - 5.64 mmol/L	*P*-value
Premenopausal women 180 (60%)				
Number (%)	80 (26.6%)	65 (21.7%)	35 (11.7%)	
Age (yr)	34 ± 10.2	35 ± 9.2	38 ± 11.3	>.05
Ca (mmol/L)	2.50 ± 0.4	2.54 ± 0.5	2.56 ± 0.9	>.05
AIP	**0.04 ± 0.01**	**0.05 ± 0.1**	**0.11 ± 0.1**	**.002** **
BMI	22.8 ± 6.2	23.8 ± 6.3	23.9 ± 6.5	>.05
Postmenopausal women 120 (40%)				
Number (%)	22 (7.3%)	50 (16.7%)	48 (16%)	
Age (year)	53 ± 9.3	54 ± 10.4	59 ± 10.2	>.05
Ca (mmol/L)	**2.50 ± 0.5**	**2.57 ± 0.7**	**2.61 ± 0.8**	**.03** *
AIP	**0.03 ± 0.01**	**0.13 ± 0.1**	**0.21 ± 0.1**	**.003** **
BMI	23.8 ± 7.1	24.7 ± 7.3	24.8 ± 8.1	>.05

Data were indicated as the mean ± (SD) or number (%) for continuous variables, *P*-value obtained from one-way ANOVA. Significant values are indicated in bold.

AIP = atherogenic index of plasma, BMI = body mass index, HDL-C = high-density lipoprotein cholesterol, LDL-C = low-density lipoprotein cholesterol.

* *P* ≤ .05.

** *P* ≤ 0.001.

Participants in Table 4 have been categorized into 3 groups according to their triglyceride levels (Normal < 1.70 mmol/L, Borderline high 1.70–2.25 mmol/L, and High 2.26–5.64 mmol/L). There was a progressive and substantial rise in serum Ca levels in postmenopausal women as triglyceride levels increased (*P* = .03). However, the levels of Ca did not exhibit any significant changes among the 3 groups of premenopausal individuals with different triglyceride levels. The AIP values were shown to be substantially associated with the risk of CVD in both pre- and postmenopausal groups with high triglyceride levels (*P* = .002 and .003, respectively).

**Table 4:** Serum calcium of women (pre- and postmenopausal) separated relating to triglyceride values.

Parameter	Normal < 1.70 mmol/L	Borderline high 1.70–2.25 mmol/L	High 2.26–5.64 mmol/L	*P*-value
Premenopausal women 180 (60%)				
Number (%)	80 (26.6%)	65 (21.7%)	35 (11.7%)	
Age (yr)	34 ± 10.2	35 ± 9.2	38 ± 11.3	>.05
Ca (mmol/L)	2.50 ± 0.4	2.54 ± 0.5	2.56 ± 0.9	>.05
AIP	**0.04 ± 0.01**	**0.05 ± 0.1**	**0.11 ± 0.1**	**.002** **
BMI	22.8 ± 6.2	23.8 ± 6.3	23.9 ± 6.5	>.05
Postmenopausal women 120 (40%)				
Number (%)	22 (7.3%)	50 (16.7%)	48 (16%)	
Age (yr)	53 ± 9.3	54 ± 10.4	59 ± 10.2	>.05
Ca (mmol/L)	**2.50 ± 0.5**	**2.57 ± 0.7**	**2.61 ± 0.8**	**.03** *
AIP	**0.03 ± 0.01**	**0.13 ± 0.1**	**0.21 ± 0.1**	**.003** **
BMI	23.8 ± 7.1	24.7 ± 7.3	24.8 ± 8.1	>.05

Data were indicated as the mean ± (SD) or number (%) for continuous variables, *P*-value obtained from one-way ANOVA. Significant values are indicated in bold.

AIP = atherogenic index of plasma, BMI = body mass index, HDL-C = high-density lipoprotein cholesterol, LDL-C = low-density lipoprotein cholesterol.

* *P* ≤ 0.05.

** *P* ≤ .001.

To account for potential confounding effects of age and BMI, we stratified the analysis by BMI categories (normal weight vs overweight/obese). AIP values and Ca levels were consistently higher in the overweight subgroups, especially among postmenopausal women. This suggests that BMI may partially mediate the relationship between Ca and lipid disturbances (Data not shown).

### 3.5. Pearson correlation analysis

**Figure 1.**
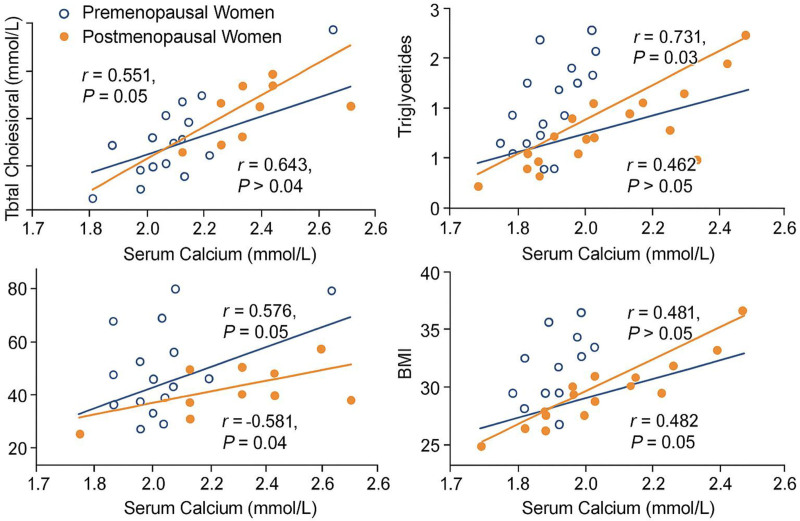
Scatter plots showing Pearson correlation between serum calcium levels and lipid/metabolic parameters in premenopausal (left column) and postmenopausal women (right column).

Moreover, the Pearson correlation revealed a statistically significant positive association between TC, TG, and serum Ca in postmenopausal women (*P* = .04, .03 correspondingly, Fig. 1, Table 5). However, a high amount of Ca was shown to have a negative link with HDL-C (*P* = .05). We found a significant link between TC and serum Ca in premenopausal women, but the correlation was smaller and positive (*P* = .05, Table 5, Fig. 1).

**Table 5:** Pearson correlation coefficient between lipids and serum calcium in pre- and postmenopausal women.

Parameter	Serum calcium (mmol/L)			
	Women premenopausal		Women postmenopausal	
	*r*	*P*	*r*	*P*
LDL-C (mmol/L)	0.332	>.05	0.441	>.05
HDL-C (mmol/L)	−0.313	>.05	−**0.513**	**.05** *
Total cholesterol (mmol/L)	0.551	**.05** *	0.643	**.04** *
Triglycerides (TG) (mmol/L)	0.462	>.05	**0.731**	**.03** *
PTH (pg/mL)	**0.576**	**.05** *	−**0.581**	**.04** *
AIP	0.422	>.05	**0.521**	**.05** *
BMI	0.482	>.05	**0.541**	**.05** *

*P*-values were obtained from Pearson correlation; Starred values point to a significant level. Significant values are indicated in bold.

HDL-C = high-density lipoprotein cholesterol, LDL-C = low-density lipoprotein cholesterol.

* *P* ≤ .05.

** *P* ≤ .00.

### 3.6. Key findings

In postmenopausal women, serum Ca showed: Positive correlation with TC (*R* = 0.643, *P* = .04); Positive correlation with TG (*R* = 0.731, *P* = .03); Negative correlation with HDL-C (*r* = −0.513, *P* = .05).
In premenopausal women: Positive correlation between Ca and TC (*R* = 0.551, *P* = .05).

## 4. Discussion

The relationship between serum Ca levels and lipid metabolism has garnered considerable interest due to its implications for cardiovascular health. Elevated serum Ca levels have been associated with adverse lipid profiles and an increased risk of CVD, particularly in postmenopausal women. This study explores the associations between serum Ca levels and lipid levels and considers the differences between premenopausal and postmenopausal women. Ca plays a crucial role in various physiological processes, including muscle contraction, neurotransmission, and intracellular signaling. Recent evidence suggests that Ca may also influence lipid metabolism through several mechanisms: Ca influences the secretion and action of hormones such as insulin and PTH. Elevated Ca levels can impair insulin secretion and action, leading to insulin resistance, a condition closely linked to dyslipidemia.^[19]^ Insulin resistance promotes the synthesis of TG and reduces HDL-C, contributing to an adverse lipid profile. Also, high serum Ca levels have been associated with increased oxidative stress and inflammation.^[20,21]^ These processes can lead to endothelial dysfunction and the development of atherosclerosis. Inflammation can also alter lipid metabolism by increasing the production of very low-density lipoprotein and reducing HDL-C levels. Ca is involved in adipocyte function and lipid storage. High intracellular Ca levels in adipocytes can enhance lipogenesis and reduce lipolysis, leading to increased fat accumulation and dyslipidemia.^[22,23]^

Ca metabolism is closely linked with Vit-D status. Vit-D deficiency, which is common in postmenopausal women, can lead to secondary hyperparathyroidism and elevated Ca levels. This can exacerbate insulin resistance and inflammation, further contributing to adverse lipid profiles.^[24,25]^ An example that supports our cases (where women who were postmenopausal had a deficiency in Vit-D) is a research study conducted by Kambal and colleagues in 2023,^[26]^ which specifically evaluated the level of Vit-D in Saudi women. The study found a significant occurrence of Vit-D deficiency, which aligns with previous studies that have linked this deficiency to factors such as limited sunlight exposure, being indoors for extended periods, and wearing veils. This may elucidate the heightened Ca levels observed in postmenopausal women within our cohort study. Our findings align with global data, such as the meta-analysis by Li et al^[11]^ (2022), which identified a consistent link between elevated serum Ca and adverse lipid profiles. A Saudi-based study by Kambal et al^[25]^ (2023) similarly highlighted widespread Vit-D deficiency in women, a key factor that disrupts Ca metabolism. In contrast, other global studies, found neutral effects of Ca supplementation on lipid outcomes. These discrepancies highlight the importance of context-specific factors, including dietary habits, sun exposure, and supplement practices unique to Middle Eastern populations.

Additionally, our study found that premenopausal women have elevated levels of PTH and nearly normal levels of Ca, while postmenopausal women have normal levels of PTH but high levels of Ca. It was also noted that 20% of premenopausal women and 80% of postmenopausal women reported regular use of Ca + vitamin C tablets without consulting a physician. Furthermore, a meta-analysis published in 2010 found a significant association between Ca supplementation and an increased risk of myocardial infarction in both males and females.^[27]^ Furthermore, an elevated serum PTH level in conjunction with high Ca levels also inhibits the activity of lipoprotein lipas. A mutation in this enzyme can result in elevated levels of TG, reduced levels of LDL-C, and reduced levels of HDL-C.^[9]^ These mechanisms elucidate the correlation between serum Ca and lipid profile in our study subjects. Several observational studies have been published that correlate dietary Ca consumption, and Ca supplementation, with CVD.^[28–30]^ However, multiple other studies have failed to establish any significant connections between Ca consumption and the likelihood of changes in serum lipid profiles.^[31]^ While this study included essential regulators of Ca homeostasis namely PTH and Vit-D we acknowledge that other hormonal and metabolic markers were not assessed. In particular, estrogen, insulin resistance indices (such as homeostatic model assessment of insulin resistance), and inflammatory markers (e.g., C-reactive protein, interleukins) play pivotal roles in the regulation of both lipid metabolism and Ca balance, especially during the menopausal transition. Estrogen deficiency is known to adversely affect lipid profiles and bone resorption, potentially contributing to elevated serum Ca levels in postmenopausal women. Additionally, insulin resistance and systemic inflammation can promote dyslipidemia and vascular calcification, thereby confounding the relationship between Ca and lipid markers. The absence of these parameters in our dataset limits our ability to fully elucidate the underlying mechanisms driving the observed associations. We recommend that future investigations incorporate these biomarkers to provide a more comprehensive understanding of the endocrine metabolic interactions influencing cardiovascular risk in women.

Furthermore, the menopausal transition is associated with significant hormonal changes that affect Ca and lipid metabolism. Understanding these differences is critical for elucidating the observed associations in our study. Menopause is characterized by a decline in estrogen levels, which has profound effects on lipid metabolism. Estrogen has a protective effect on lipid profiles, increasing HDL-C levels and reducing LDL-C and triglyceride levels.^[32]^ The decline in estrogen after menopause leads to adverse changes in lipid profiles, with increased TC, LDL-C, and TG, and decreased HDL-C levels. Our descriptive data (Table 1) showed that all parameters of the lipid profiles were within normal values in premenopausal women. However, the BMI was slightly elevated, indicating overweight. This might perhaps explain why the AIP was shown to be a risk factor for CVD in premenopausal women, with a value slightly >0.1. In contrast, postmenopausal individuals exhibited elevated levels of TC, TG, and LDL-C, while HDL-C was low. Additionally, the BMIs of all groups indicated overweight. Consequently, the AIP value was 0.11, which is linked to an intermediate risk of CVD among postmenopausal individuals. However, by not controlling the age and BMI variables, the analysis may overlook important influences that could skew the results. For instance, if postmenopausal women in the study are generally older and have higher BMI compared to premenopausal women, the differences in lipid parameters might be due in part to age and BMI rather than menopause alone. This omission can lead to confounding, where the effect of menopause on lipid levels is mixed with the effects of age and BMI, making it difficult to draw accurate conclusions about the true impact of menopause on lipid profiles.

In this study, menopausal status was determined based on participants’ self-reported menstrual history, a method commonly used in large-scale epidemiological and clinical studies due to its feasibility, cost-efficiency, and non-invasiveness. Several previous studies have demonstrated reasonable agreement between self-report and hormonal definitions of menopause, particularly when menstrual cessation exceeds 12 months.^[33,34]^ This approach allowed us to classify women efficiently within a university-based population without imposing the added financial or logistical burden of hormone testing (e.g., follicle-stimulating hormone, estradiol), which was not feasible within the scope of the study. Nevertheless, we recognize that self-report may be prone to misclassification, especially in perimenopausal women who may experience irregular cycles despite fluctuating hormonal levels. Such misclassification could attenuate the observed differences between pre- and postmenopausal groups. To mitigate this in future research, the inclusion of hormonal confirmation is recommended, particularly in studies aiming for precise subgroup delineation or mechanistic insights.

Furthermore, postmenopausal women often experience changes in Ca homeostasis, including increased serum Ca levels and a higher prevalence of hyperparathyroidism.^[35]^ These changes can exacerbate the negative effects of menopause on lipid metabolism and increase the risk of CVD. Estrogen deficiency after menopause also affects bone metabolism, leading to increased bone resorption and release of Ca into the bloodstream. This contributes to higher serum Ca levels in postmenopausal women.^[36]^ Elevated serum Ca can influence lipid metabolism and increase the risk of dyslipidemia and CVD.

Our study’s findings have several important clinical implications, particularly for the management of cardiovascular health in postmenopausal women. Elevated serum Ca levels, along with adverse lipid profiles, can serve as important biomarkers for assessing cardiovascular risk in postmenopausal women. Our findings (Tables 3 and 4) revealed a significant relationship between cholesterol and Ca levels only, in premenopausal women. On the other hand, there was a clear and highly significant relationship between cholesterol, TG, Ca, and AIP among postmenopausal women, which explains the role of increased Ca levels and the appearance of AIP among postmenopausal women as a risk factor (Table 5). Regular monitoring of lipids parameters and Ca can help identify women at higher risk of developing CVD and facilitate early intervention. The potential impact of Ca supplementation on cardiovascular health in postmenopausal women warrants careful consideration. While Ca supplements are commonly prescribed to prevent osteoporosis, their effects on serum Ca levels and lipid profiles should be monitored. Clinicians should weigh the benefits of Ca supplementation against the potential risks of exacerbating dyslipidemia and increasing CVD risk. Our data suggest that premenopausal women with elevated Ca levels may carry a latent risk for cardiovascular complications in the future, especially if BMI and AIP values continue to increase. In contrast, postmenopausal women already show an intermediate cardiovascular risk based on AIP scores and dyslipidemia. This emphasizes the importance of early lipid and Ca screening for premenopausal women and aggressive cardiovascular prevention strategies in postmenopausal women.

This study provides novel insights into the association between serum Ca levels and lipid profiles in women, particularly postmenopausal individuals. However, the findings should be interpreted with caution due to the absence of data on important confounding factors such as dietary intake, physical activity, and medication use. These variables are known to influence both Ca metabolism and lipid regulation, and their omission may affect the strength and interpretation of the observed associations.

Moreover, the cross-sectional design limits our ability to infer causality. While we observed strong and statistically significant correlations, particularly in postmenopausal women (e.g., Ca vs TG: *R* = 0.731, *P* = .03; Ca vs HDL-C: *r* = –0.513, *P* = .05), we cannot determine whether elevated Ca levels contribute to lipid abnormalities or vice versa. It is also possible that shared underlying factors, such as inflammation, hormonal imbalance, or adipokine activity, drive both outcomes.

To establish causal relationships, longitudinal cohort studies and Mendelian randomization analyses are recommended. These approaches would help disentangle the temporal sequence and biological mechanisms linking Ca and lipid metabolism, and clarify whether Ca dysregulation is a contributing factor or a consequence of lipid disturbances.

## 5. Limitations of the study

While this study provides meaningful insights into the association between serum Ca and lipid profiles among Saudi women, several limitations should be acknowledged.

First, data on important confounding factors, such as dietary intake, physical activity, and medication use, were not collected. These variables can significantly influence both serum Ca and lipid levels. For instance, dietary Ca or supplementation may alter serum Ca concentrations, while high intake of saturated fats and cholesterol is known to adversely affect lipid profiles. Likewise, regular physical activity enhances both Ca regulation and lipid metabolism. The lack of this information makes it challenging to assess the extent to which these variables contributed to the observed associations.

Second, menopausal status was determined solely based on self-reported menstrual history, without biochemical verification through hormonal assays such as follicle-stimulating hormone or estradiol. Although self-report is widely used in population studies due to its practicality and low cost, it may lead to misclassification, particularly among perimenopausal women with irregular cycles. This could potentially dilute the differences observed between pre- and postmenopausal groups.

Third, we lacked detailed information on the use of relevant medications, including lipid-lowering agents, Ca or Vit-D supplements, and hormone replacement therapy. These medications may significantly influence biochemical measurements and introduce unmeasured bias.

Fourth, while participants with known metabolic disorders or Vit-D deficiency requiring treatment were excluded, we did not explicitly screen for parathyroid disorders such as primary or secondary hyperparathyroidism. These conditions can markedly alter Ca homeostasis and may have influenced the results, particularly among postmenopausal women who already experience hormonal shifts affecting Ca metabolism. Additionally, other potentially confounding lifestyle factors such as smoking status and coexisting health conditions were not assessed.

Fifth, although our total sample size (n = 300) was determined based on a priori power analysis, subgroup analyses – especially among postmenopausal women (n = 120), may still have been underpowered to detect smaller effect sizes. Nonetheless, several statistically significant associations were observed in this group (e.g., serum Ca vs TC: *R* = 0.643, *P* = .04), indicating that the findings are likely robust.

Sixth, a key limitation is the absence of important hormonal and metabolic markers, such as estrogen, insulin resistance indices (e.g., homeostatic model assessment of insulin resistance), and inflammatory biomarkers (e.g., C-reactive protein, IL-6). These parameters play pivotal roles in modulating both Ca and lipid metabolism, particularly during the menopausal transition. Estrogen deficiency is known to worsen lipid profiles and increase bone resorption, while insulin resistance and systemic inflammation contribute to dyslipidemia and vascular calcification. Without these variables, the mechanistic interpretation of our findings remains incomplete.

Seventh, participants were recruited exclusively from a single institution, Taibah University in Madinah, which may introduce selection bias and limit the generalizability of the findings. The study population may not reflect the broader demographic, socioeconomic, or cultural characteristics of Saudi women from other regions or backgrounds. University-affiliated individuals may have better access to healthcare, different lifestyle behaviors, or higher health literacy. Therefore, caution is warranted in extrapolating these results to the general population. Future research should consider multicenter recruitment to enhance external validity and ensure broader representativeness.

Given these limitations, causal inference should be made with caution. The lack of comprehensive data on confounding factors restricts our ability to fully explain the nature of the associations observed. Future studies should aim to include detailed assessments of diet, physical activity, medication use, hormonal status, and inflammatory markers. Moreover, longitudinal and interventional research designs will be essential to clarify temporal relationships and deepen our understanding of how Ca metabolism influences cardiovascular risk, particularly in postmenopausal women.

## 6. Conclusion

This study provides novel and region-specific evidence of a significant association between elevated serum Ca levels and adverse lipid profiles, specifically increased TC and TG, in postmenopausal Saudi women, a group at heightened risk for CVD. Notably, this is one of the first studies in Saudi Arabia to examine this relationship while differentiating between premenopausal and postmenopausal women, offering new insights into how menopausal status influences Ca, lipid interactions.

A key benefit of this research lies in its potential to inform preventive screening strategies: monitoring serum Ca and lipid levels, particularly in overweight or supplement-using postmenopausal women, may offer an accessible and cost-effective approach for early identification of cardiovascular risk.

Although cross-sectional design limits causal interpretation, the study lays an important foundation for future multicenter and longitudinal research. By addressing a gap in the literature and focusing on an underrepresented population, this study supports the development of targeted, gender, and context-specific preventive interventions to improve cardiovascular outcomes among women in the Middle East.

## Acknowledgments

The authors gratefully acknowledge the financial support provided by Taibah University (Al-Madinah, Kingdom of Saudi Arabia), which enabled the successful completion of this study. The authors are grateful to Miss Johayna Aboalkayer (Taibah University, Medical Applied Science College’s technician) for her technical support.

## Author contributions

**Conceptualization:** Walaa Mohammedsaeed, Amal Mohammed Q. Surrati, Asma Alshanqiti.

**Investigation:** Walaa Mohammedsaeed, Amal Mohammed Q. Surrati, Asma Alshanqiti.

**Methodology:** Walaa Mohammedsaeed.

**Supervision:** Walaa Mohammedsaeed.

**Validation:** Walaa Mohammedsaeed, Amal Mohammed Q. Surrati, Asma Alshanqiti.

**Writing – original draft:** Walaa Mohammedsaeed.

**Writing – review & editing:** Amal Mohammed Q. Surrati, Asma Alshanqiti.

## References

[1] Yang C Shi X Xia H. The evidence and controversy between dietary calcium intake and calcium supplementation and the risk of cardiovascular disease: a systematic review and meta-analysis. J Am Coll Nutr. 2020;39:352–70. 31625814 10.1080/07315724.2019.1649219

[2] Heshmati J Sepidarkish M Namazi N. Impact of dietary calcium supplement on circulating lipoprotein concentrations and atherogenic indices. J Diet Suppl. 2019;16:357–67. 29561197 10.1080/19390211.2018.1440685

[3] Hajhashemy Z Rouhani P Saneei P. Dietary calcium intake and type 2 diabetes: a meta-analysis. Sci Rep. 2022;12:1050. 35058558 10.1038/s41598-022-05144-8 PMC8776796

[4] Shin SK Kim MK Lee YH. Dietary calcium intake and metabolic syndrome in rural Korea. Nutr Res Pract. 2015;9:328–35. 26060546 10.4162/nrp.2015.9.3.328 PMC4460066

[5] Zemel MB. Role of dietary calcium and dairy products in modulating adiposity. Lipids. 2020;38:139–46. 10.1007/s11745-003-1044-6 12733746

[6] Kunutsor SK Whitehouse MR Blom AW Laukkanen JA. Serum calcium levels and cardiovascular disease risk. Eur J Epidemiol. 2017;32:593–603. 28405867 10.1007/s10654-017-0242-2 PMC5570773

[7] Jørgensen HL Nordestgaard BG Zacho J. Serum calcium and risk of ischemic heart disease and myocardial infarction: a Mendelian randomization study. J Clin Endocrinol Metab. 2022;107:e1146–55.

[8] El Khoudary SR Greendale GA Crawford SL. Menopause transition and cardiovascular disease risk: evidence from the SWAN study and other cohorts. Circulation. 2020;141:556–68.

[9] Reid IR Bolland MJ Avenell A. Effects of calcium supplementation on serum lipids: a randomized controlled trial and meta-analysis. J Clin Endocrinol Metab. 2017;102:3824–34.

[10] Wimalawansa SJ. Vitamin D deficiency: oxidative stress and aging. Biology (Basel). 2018;8:30. 10.3390/biology8020030 PMC6627346 31083546

[11] Li M Zhao H Ma Y. Association between serum calcium levels and cardiovascular disease in US adults: the NHANES 2005–2016. Front Cardiovasc Med. 2022;9:910840.

[12] Varbo A Nordestgaard BG Benn M. Triglyceride-rich lipoproteins, high-density lipoprotein cholesterol, and risk of ischemic heart disease. Eur Heart J. 2021;42:285–95.33410466

[13] Grundy SM Stone NJ Bailey AL. 2018 AHA/ACC cholesterol guidelines. Circulation. 2019;139:e1082–143.30586774

[14] Mach F Baigent C Catapano AL; ESC Scientific Document Group. 2019 ESC/EAS guidelines for lipid modification. Eur Heart J. 2020;41:111–88. 31504418 10.1093/eurheartj/ehz455

[15] Bouillon R Antonio L Olarte OR. Comparative analysis of vitamin D deficiency treatment guidelines. Nutrients. 2022;14:1168.35334824

[16] KDIGO Clinical Practice Guideline for CKD–MBD. Kidney Int Suppl. 2024;14:1–91. 10.1038/ki.2009.188 19644521

[17] Wekesa C Asiki G Kasamba I. Atherogenic risk in rural Uganda. J Trop Med. 2016;2016:7073894. 27418933 10.1155/2016/7073894 PMC4933868

[18] World Health Organization (WHO). Body mass index (BMI) classification. WHO; 2022. https://www.who.int/news-room/fact-sheets/detail/obesity-and-overweight. Accessed October 1, 2025.

[19] Xu L Lin SL Schooling CM. Effect of calcium on coronary artery disease: Mendelian randomization. Sci Rep. 2017;7:42691. 28195141 10.1038/srep42691 PMC5307362

[20] Chen JM Wu TY Wu YF Kuo KL. Serum calcium and metabolic syndrome. Arch Endocrinol Metab. 2023;67:e000632. 37249460 10.20945/2359-3997000000632 PMC10665046

[21] Hua Y Liu HL Sun JY Kong X-Q Sun W Xiong Y-Q. Serum calcium and hypertension among US adults. Front Cardiovasc Med. 2021;8:719165. 34912855 10.3389/fcvm.2021.719165 PMC8666532

[22] Tarran R. Calcium signaling in adipocytes and metabolic regulation. Nat Metab. 2023;5:125–36.

[23] Haider F Ghafoor H Hassan OF Farooqui K Bel Khair AOM Shoaib F. Vitamin D and cardiovascular diseases: an update. Cureus. 2023;15:e49734. 38161941 10.7759/cureus.49734 PMC10757591

[24] Artaza JN Contreras S Garcia LA. Vitamin D and cardiovascular disease: disparities. J Health Care Poor Underserved. 2011;22:23–38. 22102304 10.1353/hpu.2011.0161 PMC3417128

[25] Kambal N Abdelwahab S Albasheer O. Vitamin D knowledge among Saudi women. Medicine (Baltim). 2023;102:e36529. 10.1097/MD.0000000000036529 PMC10735156 38134098

[26] Chun S. Calcium intake and myocardial infarction: meta-analysis. Nutrients. 2022;14:1785.35565753

[27] Van Hemelrijck M Michaelsson K Linseisen J Rohrmann S. Calcium intake and cardiovascular death. PLoS One. 2013;8:e61037. 23593383 10.1371/journal.pone.0061037 PMC3622603

[28] Michaelsson K Melhus H Lemming EW. Long-term calcium intake and mortality. BMJ. 2013;346:f228. 23403980 10.1136/bmj.f228 PMC3571949

[29] Moore-Schiltz L Albert JM Singer ME Swain J Nock NL. Calcium and magnesium intake in metabolic syndrome. Br J Nutr. 2015;114:924–35. 26259506 10.1017/S0007114515002482

[30] Aritici G Bas M. Metabolic syndrome and calcium in premenopausal women. Prog Nutr. 2018;20:220–8.

[31] Kim H Kim JH Lee JE. Association between osteoporosis and cardiovascular disease in postmenopausal women: a Korean nationwide study. Bone. 2021;147:115929.33737192

[32] Harlow SD Gass M Hall JE; STRAW+10 Collaborative Group. Executive summary of the stages of reproductive aging workshop +10: addressing the unfinished agenda of staging reproductive aging. Climacteric. 2012;15:105–14. 22338612 10.3109/13697137.2011.650656 PMC3580996

[33] Gold EB Sternfeld B Kelsey JL. Demographic and lifestyle factors in midlife women. Am J Epidemiol. 2000;152:463–73. 10981461 10.1093/aje/152.5.463

[34] Harlow SD Gass M Hall JE; STRAW+10 Collaborative Group. STRAW+10: addressing reproductive aging. Climacteric. 2012;15:105–14. 22338612 10.3109/13697137.2011.650656 PMC3580996

[35] Tankó LB Christiansen C Cox DA Geiger MJ McNabb MA Cummings SR. Osteoporosis and cardiovascular disease in postmenopausal women. J Bone Miner Res. 2005;20:1912–20. 16234963 10.1359/JBMR.050711

[36] Sambrook P Cooper C. Osteoporosis. Lancet. 2006;367:2010–8. 16782492 10.1016/S0140-6736(06)68891-0

